# Connecting to Cognition: A Methodological Journey With Urban Dementia

**DOI:** 10.1177/13607804241287683

**Published:** 2025-05-22

**Authors:** James Rupert Fletcher

**Affiliations:** University of Bath, UK

**Keywords:** analysis, creative, digital, go-along, mapping, representation

## Abstract

Contemporary sociological developments spanning the ontological turn, post-cognitivism, biosocial transdisciplinarity, post-qualitative inquiry, and new empiricism are opening up novel sites of methodological contention regarding the nature of cognition as a research object. While these developments are conceptually stimulating, their (potential) overlapping methodological implications are under-explicated. Outlining a moving ethnography of urban transport use by passengers with dementia, in this article I delve into the more pragmatic methodological contingencies of attempts to engage with cognition as a fundamentally ecological phenomenon. Blurring the boundaries between the management and analysis of data, I develop a laborious and meditative ‘journeying analysis’ as a means of dealing with an unruly dataset. In line with post-qualitative commitments, I pursue a somewhat haphazard approach to anti-representation, while nonetheless embracing the researcher-centrism of doing research. I advocate creative digital strategies – for example, map-making, soundscaping – for cultivating data forms that offer multiple possibilities for audiences to forge new connections with the study phenomena.

## Introduction

This article presents a methodological contribution to contemporary sociological interest in urban mental health. More ambitiously, it speaks of the broader problem, provoked by such interest, of how we can practically cultivate sociological engagements with cognition and environments, two subjects that might traditionally be considered the rightful intellectual property of cognitive science and geography. Importantly, as will become clear, I am not talking about how thought and place relate, interact, co-determine, and so on. Rather, I am proposing that, vis-a-vis a sociology of ecological connection, cognition and environment can be fruitfully approached as the same thing. Moreover, I do not solely mean ‘approached’ here as ‘conceptualised’, ‘theorised’, or otherwise ‘thought about’. I am specifically interested in how that thinking about might correspond with real methodological engagements, and vice versa, how that methodological engagement might correspond with thinking about thinking.

Psychiatric epidemiology has long documented associations between urbanicity and mental disorder, classically positing that the former effects the latter, or that the latter effects the former, or even both at once (Pignon et al., 2023). In response, sociologists have criticised this epidemiological work for too crudely engaging with urban environments as merely a collection of ‘environment’ variables. Even when urbanicity is expanded upon, the resulting characterisations are repeatedly stereotype-laden, for example, the city is inherently hostile, so that it becomes almost obvious that such negative circumstances would be associated with correspondingly negative modes of psychic life (Fletcher and Birk, 2020). Exemplifying the need to take urban environments more seriously as complex social, cultural, political, and economic ecologies, simultaneously offering immense peril and promise, perhaps the most influential sociological interjection in urban mental health is Klinenberg’s (2015) study of the 1995 Chicago heat wave. This work showed the potent capacities of cities to constitute human health, including psychiatric, via their catalysation of many forces, for example, climate change, racism, capitalism, and so on. There was far more at stake in cities such as Chicago than merely a ‘hostile environment’ variable that determined inhabitants’ outcomes.

While Klinenberg’s original study was trailblazing, subsequent years have played host to resurgent biosocial sociologies, seeking greater transdisciplinary engagement with the cognitive sciences as a means of enhancing conceptual and empirical approaches to the blending of inner and outer human worlds. Today, a growing sociological scholarship attends to urban psychiatric disorder as a ‘node of the biosocial and biopolitical everyday’, wherein the cerebro-cognitive, sociocultural, and politico-economic become ever more difficult to disentangle (Rose and Fitzgerald, 2022: 3). Nonetheless, to date, that ‘cerebro-cognitive’ facet floats comparatively freely of sociological inquiry, as something patched onto our thinking from elsewhere (Fletcher and Birk, 2022). In this article, I suggest that this need not be our lot, and show how we might move further towards studying urban psychiatric disorder, and emplaced cognitive life more broadly, as authentically, ontologically, and radically sociological.

## Radicalising cognition

Since the 1990s, a small subfield of cognitive sociology has developed in semi-relation with cognitive science. Although peripheral to cognitive science and its dominant traditions of cognitive neuroscience and cognitive psychology, cognitive sociology has nonetheless been remarkably compliant with core commitments to cognitivism (Fletcher, 2024a). Cognitivism has dominated the conceptualisation of cognition since the mid-20th century, imagining the brain as a biomolecular supercomputer, with different regions performing discrete information-processing tasks. There are various neuroscientific and psychological flavours of this approach, leaning more towards the brain and mind, respectively, but they share an underlining cognitivist disposition. This understanding of cognition owes much to the high-profile development of computer science during the mid-20th century, with computer scientists likewise depicting early computers as mechanical brains, hence the longstanding prevalence of computing analogies in cognitive science (Paul, 2021). Inspired by 1960s constructionism and the idea that knowledge itself is socially constructed, cognitive sociology has typically taken cognitivism for granted and built sociological subject matter on top of the cognitioning brain and/or mind. While various sub-genres of cognitive sociology have branched out, all broadly posit forms of social determination that influence the underlying and intrinsic phenomenon of cognition (Brekhus, 2015). Hence, the ‘cognitive’ very much trumps the ‘sociology’.

As I have argued elsewhere (Fletcher, 2023b, 2023c, 2024a), two developments offer potential routes beyond conventional cognitive sociology and its relationship with mainstream cognitive science. The first comes from the outskirts of cognitive science itself: what I broadly define as post-cognitivist cognitive science. This scholarship encompasses a mass of distinct research interests, but generally coalesces around the argument that cognition occurs beyond the brain and/or mind. Some researchers point to embodiment, emphasising pre-reflective action and the processing of information via sensorimotor capacities (Shapiro, 2019). Others highlight the distribution of cognition to objects such as smartphones, cochlear implants, and walking canes (Favela et al., 2021) and to other people in social groups, whereby information processing is system-contingent rather than individual-contingent (Tollefsen, 2015). At the extreme, radical enactivists reject mental representation outright, arguing that cognition is emergent in dynamic systems, generated by complex interactions without being attributable to any individual component (Ward et al., 2017). For example, consider the way a slinky navigates stairs (Figure 1), neatly executing a complicated task solely via the physical properties of itself and its environment, and the relations between the two. The second useful development for challenging cognitivism-centred sociologies is the recent ontological turn across the social sciences and humanities. From the 2000s onward, this ontological turn has troubled sociological predilections for both materialist realist and constructivist anti-realist ontologies. Combining the eclectic influences of non-European philosophy and quantum physics, the ontological turn has foregrounded flat and monist ontologies, variably casting relations-between-things as the basic unit of reality (Gullion, 2018; Pickering, 2017). Corresponding new materialist sociologies are increasingly popular and have pronounced, but as yet unrealised, affinities with post-cognitivist cognitive science (Fletcher, 2023b, 2023c, 2024a).

**Figure 1.** Slinky slinking.

In response, I have argued for more ontologically forthright cognitive sociologies, engaging with cognition as an inherently and fundamentally sociological entity, as opposed to something that is secondarily influenced by social determinants (Fletcher, 2024a). By this, I mean that post-cognitivist conceptualisations, such as radical enactivism, whereby cognition is a process of material interactions, could be comprehensively accounted for within some contemporary monist ontologies. Consider, for instance, a 21st-century monist ontology taken from the sociology of personal life: ‘connective ontology’ (Fletcher, 2023a; Mason, 2011). Connective ontology posits the world is made up of connections themselves rather than the things that are connected. These connections are ineffable energies that emanate in dynamic ‘ecologies’, encompassing people, possessions, places, animals, technologies, climates, and all the other components of a situation that in relation make up ‘the sway of life itself’ (Mason, 2018: 125). Ecologies are irreducible worldings, animating and animated by the connections flowing through them, rendering those connections potent through a sort of processual vitalism (e.g. Greco, 2021). Conceptualising cognition as connection vis-a-vis connective ontology offers an example of how sociology might bring post-cognitivism and the ontological turn to bear on issues that have historically been forfeited to the cognitive sciences. Here, cognition can be understood as a connective energy emanating in ecologies, a force animated by dynamic relations that is essentially part of those ecologies (Fletcher, 2024a).

These conceptual developments hint at scintillating new sociological routes into urban psychiatric disorder. However, they leave us with a lot of methodological questions regarding how we actually go about studying this stuff. Indeed, this introduction exemplifies perhaps the most substantial problem running through cognitive sociology, post-cognitivist cognitive science, and sociology’s ontological turn. We enjoy a glut of theory, yet little empirical realisation of that rich conceptual promise. To this end, Ignatow (2014: 990) argues of cognitive sociology: ‘We need to show how what we do contributes to sociological methods, and not only say that what we do contributes to sociological theory’. Similarly, Parada and Palacios-García (2022) contend that across much post-cognitivist work, ‘empiricism has lagged somewhat behind regarding the embracement of paradigmatic changes’. This article is an attempt to tackle the empirical problem, and the various calls and critiques surrounding it, head on, by outlining an empirical approach to the generation and analysis of data that are tailored to connective cognition. Focussing in on ecological energies, I want to work through how we might get at cognition that is more like a slinking slinky and less like a computing computer. This matters because worthwhile sociological research on our mental lives in the complex circumstances of human life needs methodological practices that help researchers get beyond constructionism and social determinants, and thereby move closer to the core phenomena. The article is not intended as a how-to guide (though by all means, replicate any or all of it), but as a provocation to engage with methodology in a theory-heavy space.

## The study

This article reports on the Wellcome Trust [Grant: 222193/Z/20/Z] funded IN-CITU (*Interactions between Cognitive Impairment and Transport in Urban Ecologies*) project, an ethnography of dementia and transport that partly sought to address to the aforementioned methodological issues, and hence move closer to connective cognition. At a basic level, the study documented the experiences of passengers with dementia as they navigated urban public transport infrastructure. This is important because transport has major implications for people with dementia, who often lose driving licences and can easily become isolated (Fletcher, 2024c). Conceptually, the study sought to explore how connective cognition emanated in complex ecologies by focussing on people for whom everyday manifestations of cognition can be especially significant (people with dementia) and ecologies typically characterised by intense dynamic complexities (urban public transport).

The study took place in Greater Manchester, a metropolitan region of almost three million people located in the Northwest of England. Following late-20th-century deindustrialisation and decline, Greater Manchester has undergone rapid redevelopment during the 21st century. Latterly, Manchester has become one of Europe’s fastest growing cities (Deloitte, 2018), with expanding university, media, and technology sectors, a population increasing at twice the UK average (MCC, 2020) and almost 700% more per capita skyscraper construction than London (CTBUH, n.d.). Amid this re-urbanising tumult, local government is embarking on a major programme to re-municipalise public transport, bringing fragmented private services under public control for the first time anywhere in the UK since major privatisation and deregulation in the 1980s (TfGM, 2022). Furthermore, Greater Manchester became the UK’s first age-friendly city region in 2018 (Yarker and Buffel, 2022), within which, the local authority is now ‘working to make Greater Manchester the best place to live if you have dementia’ via its dedicated Dementia United government body, including the pursuit of ‘dementia-friendly’ transport (Dementia United, n.d.).

In this lively context, the study was conducted through the entirety of 2023, coinciding with the first tranche of re-municipalisation. Throughout the year, I accompanied eight passengers (Table 1) on 17 journeys, covering 131 miles in 17 hours of travel. Participants had formal dementia diagnoses, lived in Greater Manchester and regularly used public transport. They were recruited with the assistance of Dementia United (*n* = 2), the Greater Manchester Mental Health NHS Foundation Trust (*n* = 4) and word of mouth (*n* = 2). Project advertisements were handed to potential participants by the listed organisations, and with their consent the contact information of interested parties was given to me. I assessed capacity to consent during preliminary meetings at participants’ homes, a legal requirement for conducting research with people with cognitive impairments in England (Fletcher, 2023c). Procedural ethical approval was granted by the NHS North West – Greater Manchester South Research Ethics Committee [Reference: 22/NW/0142].

**Table 1.** Participants.

Pseudonym	Age	Diagnosis	Borough	Journeys	Mode
Paul	63	Vascular dementia	Bolton	1	Bus
Barbara	71	Alzheimer’s disease	Salford	6^a^	Bus/tram
Judy	82	Alzheimer’s disease	Salford	2^a^	Bus/tram
Stanley	86	Alzheimer’s disease	Manchester	2	Bus/tram
Marilyn	80	Vascular dementia	Rochdale	1	Bus
Liz	75	Mixed dementia	Bury	1	Tram
Joyce	76	Vascular dementia	Manchester	5	Bus
Nicole	80	Alzheimer’s disease	Stockport	1	Bus

a Both Judy’s journeys included Barbara.

**Figure 2.**
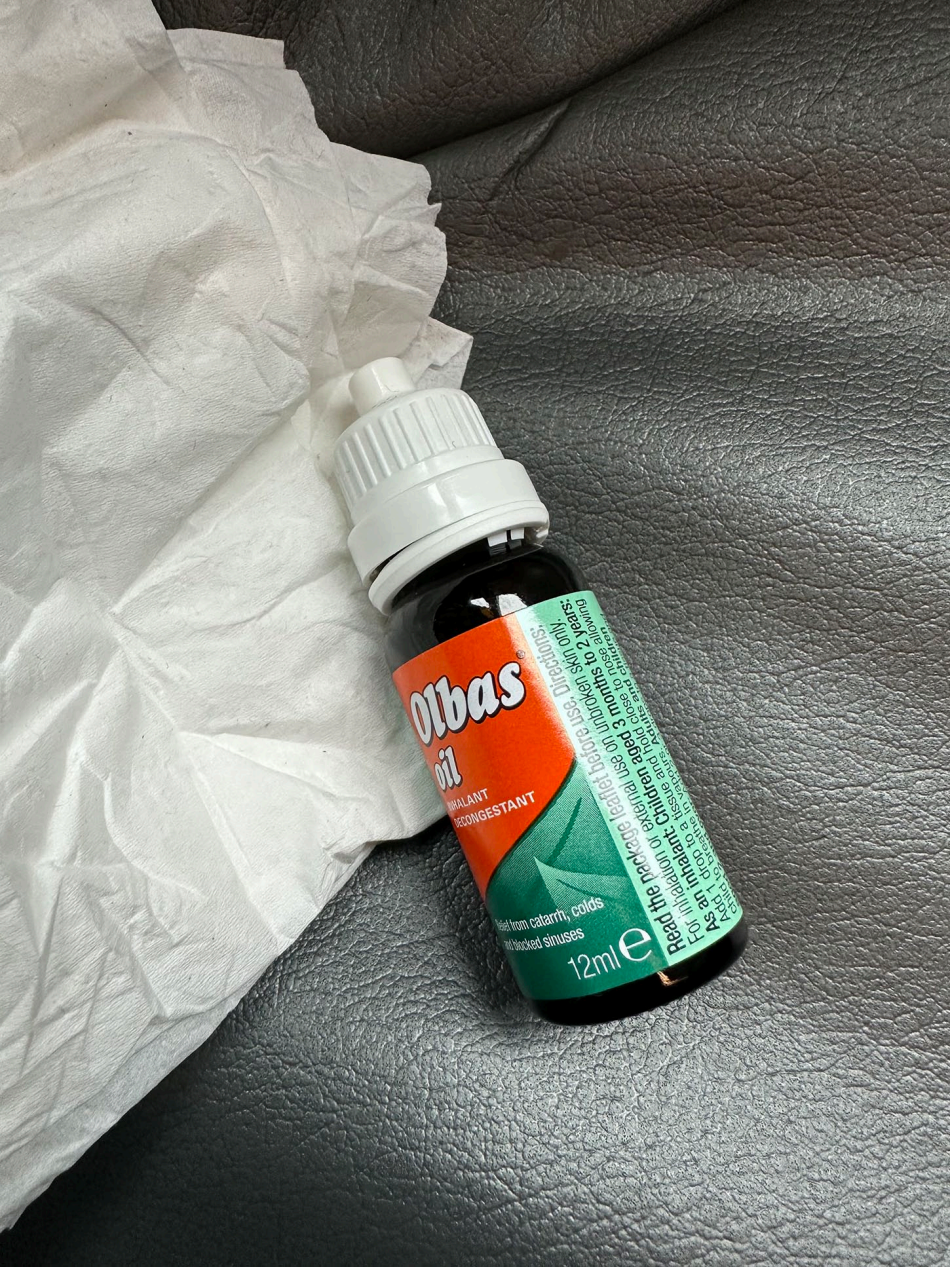
Olbas oil.

The project developed a creative go-along ethnography, accompanying participants on their usual journeys and using a range of methods to document the trips, including field notes, interviewing, audio recording, photography, videography, and geolocating. I will discuss each of these in turn below. Creative methods have peculiar ethical implications for studying cognition. Over recent decades, dementia research has moved from the widespread exclusion of people with dementia towards increasing inclusion – from participation in interviews to consultation and co-design, and now with people with dementia running their own projects (Fletcher, 2021, 2024b). In this context, I have typically shied away from purely observatory dementia research, because there is something about the politics of watching and interpreting a person with cognitive impairments, without meaningful recourse to capturing their relational expression, that does not sit easily with me. However, creative ethnography might permit more agentic forms of contribution. In particular, creative methods have considerable potential for furthering inclusive study with people with cognitive impairments by offering a variety of means for participant action, expression and meaning generation (Dowlen and Fleetwood-Smith, 2024). For instance, a person with aphasia, who might struggle to answer an interview question about the atmosphere on a bus, can take a picture of the inhalant decongestant oil (Figure 2) that she always carries with her to evoke some of the corresponding sensation of unpleasant odours.

Entering the study, I took the deliberate position that an ethnography of connective cognition required a commitment to spontaneous immersion in ecologies. Therefore, my initial disposition towards my ecologies of interest was unusually unintentional when compared with my previous experiences of conducting more conventional sociological research. To some extent, I was relatively unprepared, and deliberately so. By this I mean that I wanted to be open to allowing participants to dictate the generation of data, seeing what would happen as I accompanied people on their journeys. There are, however, big caveats to this approach. First, I do not want to fall into some naïve realism, whereby a lack of preconception allows the *real* data to speak for itself in a more natural or authentic form (this is not grounded theory; I am yet to experience data speaking for itself). As has long been noted, social researchers cannot escape their own influence in research encounters, and from a new materialist perspective this is true of everything that exists via processes of intra-relation, however conceived (Petersen, 2018). Second, there are institutional limits to unpreparedness when doing sociological research. Suffice to say that I would have neither the funding nor the ethical approvals required to do such work had I not submitted detailed proposals to various organisations. Hence, this was a heavily prepared unpreparedness.

## All aboard

Having outlined my broad approach, it is important to clarify what the different data formats offered in this study. Beginning with audio, this was recorded using PicoMic 2 Pro lapel microphones (https://www.picogear.com/store/picomic-2-pro-dual-wireless-kit/), one attached to me and the other to the person with dementia. One might assume that these microphones were dedicated to recording the verbal exchanges that made up our go-along interviews, with me asking questions and the person with dementia responding accordingly. This is partly true, inasmuch as that sometimes happened. However, the microphones and their generation of audio data were also important in their capacity to record soundscapes, broadly conceived (Figure 3). A lapel microphone is typically not far from the wearer’s ears. This means that, at least positionally, its situation is relatively proximate to that of the wearer in relation to the soundscape. The brand of microphones used in this study also had the benefit of being highly discrete, so that their presence was less likely to influence the research ecologies, compared with bulkier recording devices. Hence, while accurate recordings of participant articulations are a crucial insight into their connections, it is equally important to this study that those articulations are captured within their auditory contexts, and equally important that those soundscapes themselves are documented, because they can do their own kind of work. For instance, when listening to recordings of a bus journey, one comes to appreciate how much noise one successfully blanks out in-vivo, and can perhaps thereby gain some insight into the challenges faced by people with different sensory experiences.

**Figure 3.** Journey soundscape.

**Figure 4.**
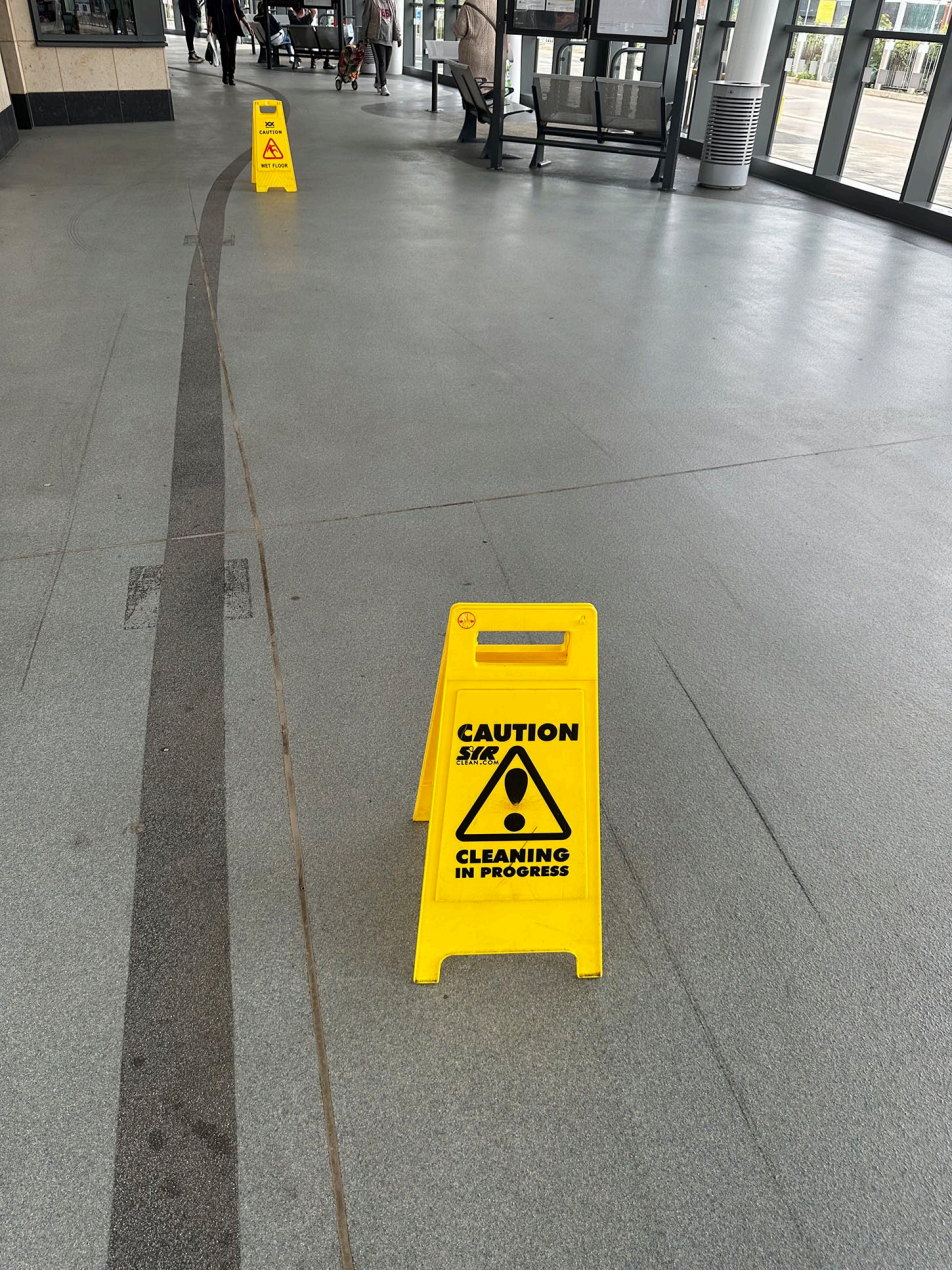
Wet floor signs.

**Figure 5.**
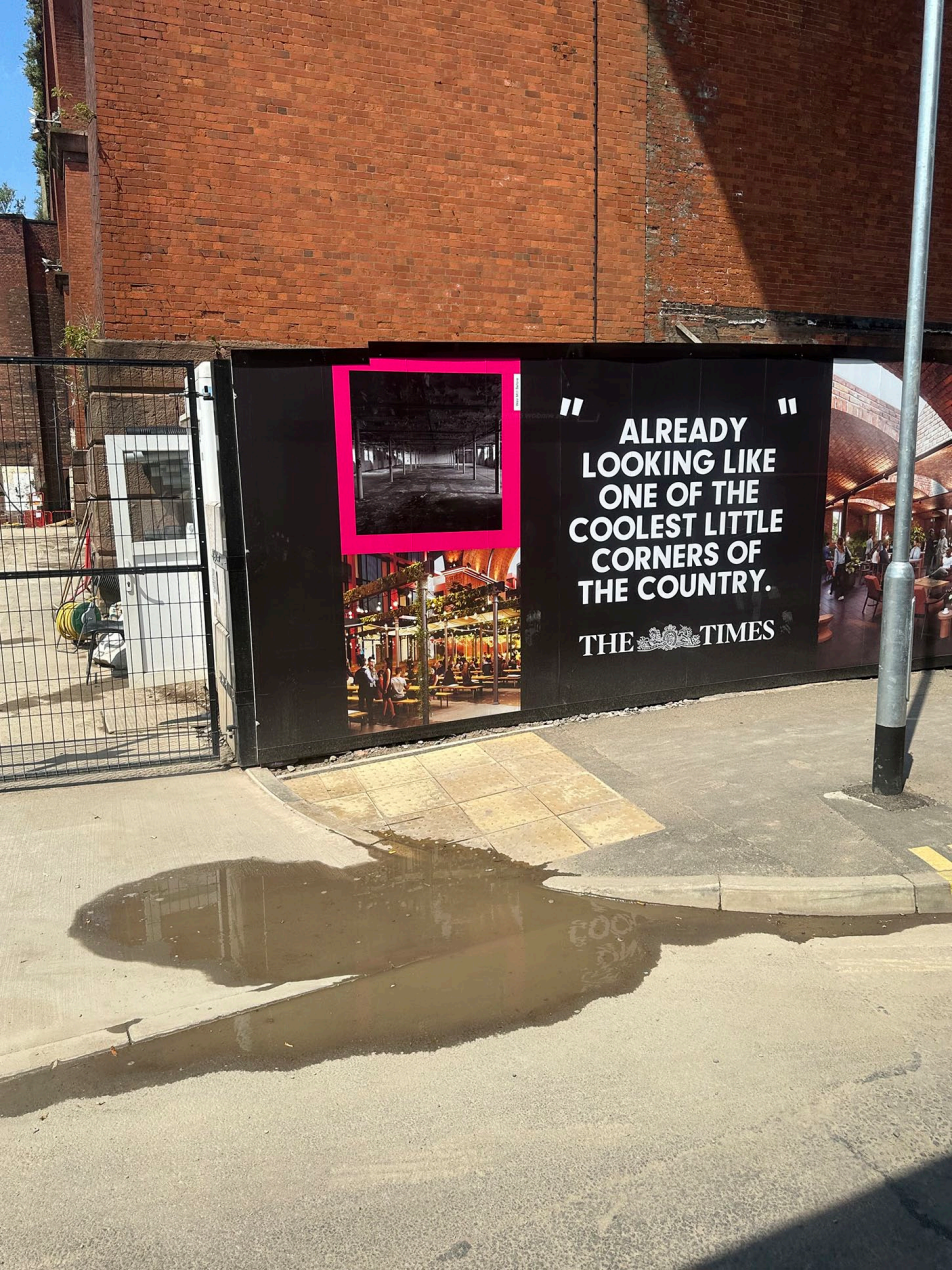
Regeneration advertisement.

**Figure 6.**
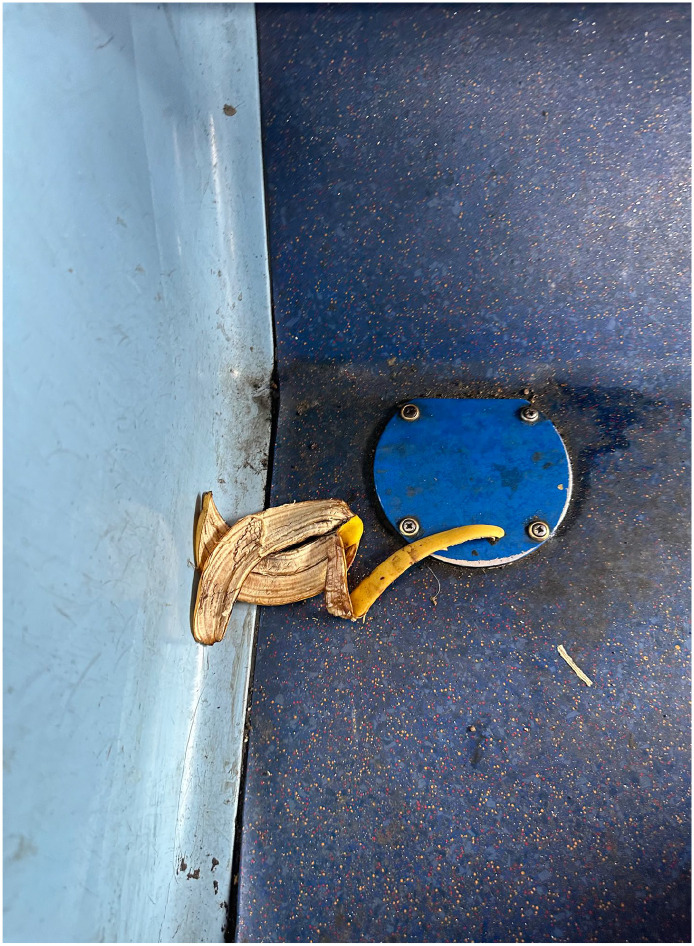
Discarded banana skin.

**Figure 7.**
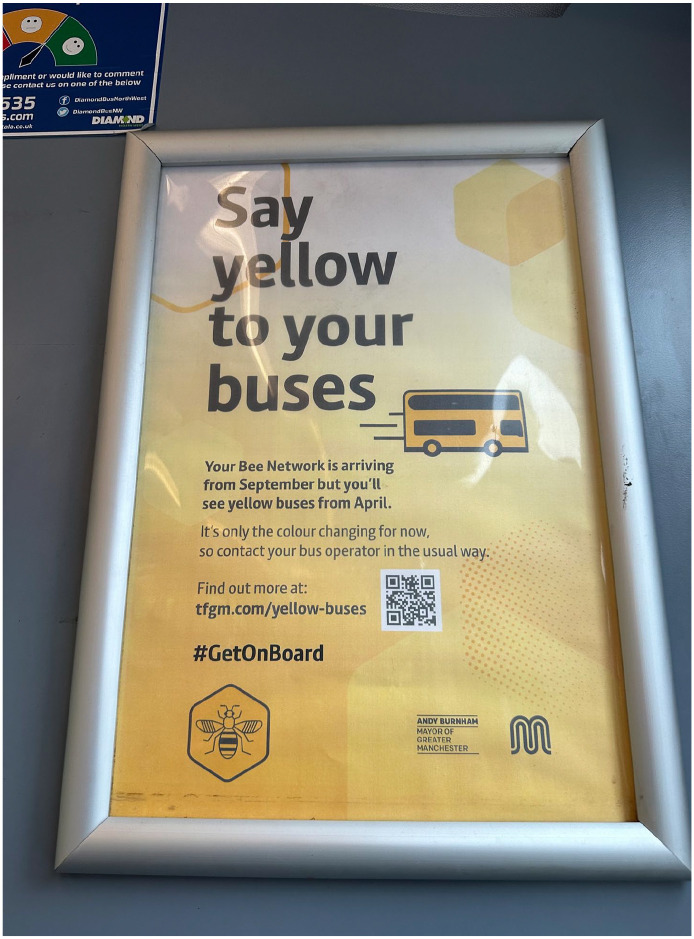
Re-municipalisation advertisement.

As well as audio recording the journeys, I photographed and videoed them, and encouraged participants to do the same (Figures 4–9). Again, partially at the request of the research ethics committee, I sought to minimise the influence of the recording equipment on the ecologies by using an iPhone 14 Pro (https://www.apple.com/go/2022/iphone-14-pro/), a popular mobile phone that one might expect to see aplenty during a normal public transport journey. Having completed the research, I can validate this expectation. Using a phone, as opposed to a DLR camera, inevitably sacrificed image quality to some extent. I deemed this to be a reasonable trade-off given that the quality of the imagery (strictly in a technical sense) is not so important as the imagery itself, its content, generation, and context. Moreover, we are talking about tiny margins of quality, as cinema quality films have now been made using the iPhone 14 Pro. The phone also had the benefit of facilitating media geolocation. As someone who captures very few photographs or videos, I prepared for data collection by signing up to Instagram to encourage me to immerse myself in an everyday manifestation of one of the core practices of my research. This proved effective. I took a lot of photos and videos of the city, spent a lot of time thinking about imagery and its generation, and a lot more time talking to people about it. During the project, I found that photographs were useful in abstracting moments – brief flashes of a facet of an ecology that seem to isolate and hence intensify it. Videos were quite different in terms of their capacity to abstract a broader sense of the ecology in process, replete with sound, motion, and a sense of time. The videos were messier and, in my opinion, less immediately evocative than the photos. They hence provided something altogether distinct, perhaps more akin to the tumultuous immersivity of urban transport itself.

**Figure 8.** Bus stop.

**Figure 9.** Viaduct view.

The use of audio-visual media can be misleading because we often intuitively engage with it as though it is the definitive record of a particular ecology. I would argue that it is important for researchers to resist the temptation to use media in this way, labouring under false ideals of unadulterated authenticity. From the standpoint of attempting to capture and convey the ecology of, for instance, an exchange in a shop, there is no inherent reason that a video of the occurrence should be a superior representation of it than a poem inspired by it. As a well-known example, I feel that Van Gogh’s painting *The Starry Night* is more ‘like’ a starry night sky than a photograph is. To me, it seems somehow more like a starry night than a starry night is itself. What I mean is that something about its artistry brings me closer to the subject matter than a more visually loyal depiction might. I am no art connoisseur, but it works on me as in inexpert audience because it has a conjuring effect. This was my approach to audio-visual media in this study. Its rationale was to resonate with and later evoke ecological connections, rather than to straightforwardly document and display.

Importantly, imagery often also represents its makers (Berger, 1972). The more curmudgeonly among us might have experienced, with puzzlement, the deliberation that some people dedicate to creating an Instagram photo. The thing being imaged is almost less important than the manipulation of an imagined audience’s sentiment towards the image. The maker can also be manifest in the image less consciously. In the film *Love Actually*, Keira Knightley discovers that Andrew Lincoln’s video of her wedding focusses exclusively on her, inadvertently revealing his love for her. Our continual embeddedness in complex ecologies typically demands selectivity – the privileging of certain connections – and a photograph has the potential to freeze an instance of that selectivity and thereby intensify it. This capacity for images to evoke something of what is behind the camera as well as in front of it renders image-making a valuable means of getting at the person and their connections. My overarching point here is that, for several reasons, multimedia should not be limited by notions of authentic documentation.

**Figure 10.**
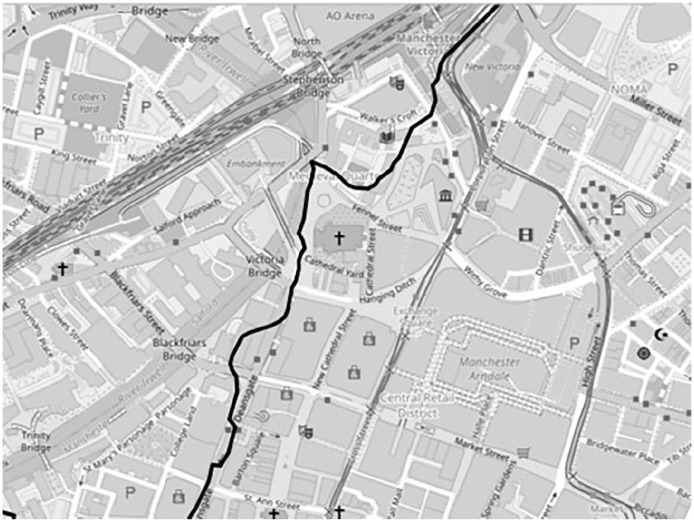
Participant getting lost.

**Figure 11.**
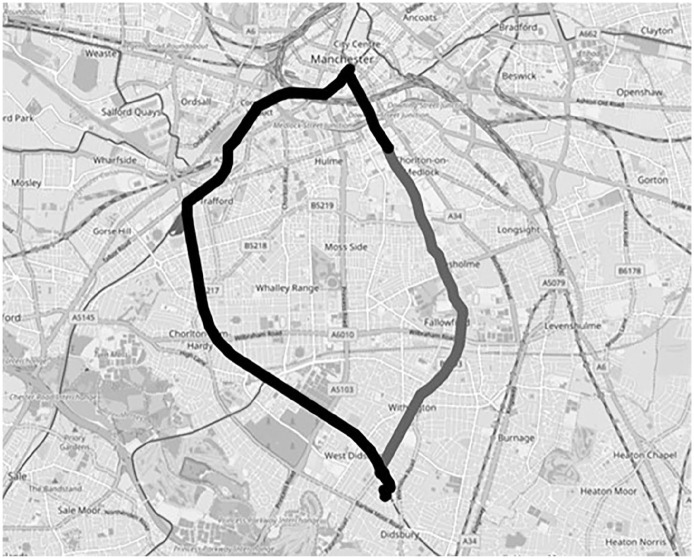
Second circuitous route.

Beyond the audio-visual data described so far, I also generated geolocation data during the journeys, recorded by the project mobile phone using the Komoot navigation app to produce GPX route location files. I then visualised these files on OpenStreetMap using the GPX.studio editing app, which offers a real-time slide-through display function to enable a digital retravelling of the journey. This data format differed from that discussed above because I primarily used it as something more akin to metadata for the rest of the dataset. In this form, it provided a context for the rest of the data, forming basic visual maps onto which all the other data formats were located. This might intuitively seem to contextualise the dataset geographically, and to some extent it did, but in practice one rarely experiences ecologies from such a perspective, that is, as a winding line covering several miles. Geolocation data was more useful as a means of contextualising the dataset in terms of time, procession, and motion. As one progresses through the final dataset, the mapped-out nature provides a sense of journeying with the data (discussed below). Hence, while traditional dataset metadata might contextualise the participant or the interview, geolocation metadata contextualised the ecologies more broadly. I suspect that the capacity for geolocation to evoke time, process, and motion is partly attributable to the recent popularisation of navigation apps, which have become a ubiquitous feature of many people’s lives via Google Maps, Strava, and so on. Geolocation data can also tell stories. In a couple of examples discussed in detail here (Fletcher, 2015), route maps themselves became potent visualisations. One jagged route represented a participant getting lost (Figure 10). Another captured a participant making a circuitous second journey to avoid problems encountered during the first journey (Figure 11).

## Journeying analysis

The amount of material that the study generated was overwhelming. Each journey produced a rich dataset of geolocation (.gpx), geolocation visualisation (.png), audio (.mp3), photographs (.jpg), videos (.mp4), transcript, and fieldnotes (.doc). This quickly posed the question of what to do with it all – how to manage it, engage with it, and analyse it in a fruitful manner. My eventual answer was a sort of ‘journeying analysis’. The first step was to catalogue everything as meticulously as possible, implementing a preset data-management filing system with a dedicated key, assigning unique identifiers to capture the interrelatedness of different data forms (Table 2). As laudable as this may appear, my strategy was primarily so laborious and meticulous as a means of pulling me through that initial overwhelmingness of the scary dataset. An important component of this data-management process was the transcription of interviews. I am a strong advocate of transcribing interviews, not as an unavoidable prelude to coding, but simply as an unparalleled (in my experience at least) means of nurturing closeness, attentiveness, and familiarity with data. It is one of those distinctly meditative tasks that is often tediously repetitive, yet demands concentration, nonetheless. I recognise that self-transcription is a luxury of having a full-time three-year personal research grant. Nonetheless, I recommend it where possible for its capacity to cultivate greater affinities with one’s data, and regret the role that the contemporary political economy of research plays in incentivising the outsourcing of transcription.

**Table 2.** Key.

Identifier				Description
P01				Participant 1
	P01j001			Participant 1, Journey 1
		P01j001k		Participant 1, Journey 1, gpx file
		P01j001g		Participant 1, Journey 1, gpx visualisation
		P01j001a		Participant 1, Journey 1, complete audio
			P01j001a001	Participant 1, Journey 1, audio clip 1
		P01j001t		Participant 1, Journey 1, complete transcript
			P01j001a001t	Participant 1, Journey 1, audio clip 1 transcript
		P01j001n		Participant 1, Journey 1 notes
			P01j001p001	Participant 1, Journey 1, photograph 1
			P01j001v001	Participant 1, Journey 1, video 1

**Figure 12.**
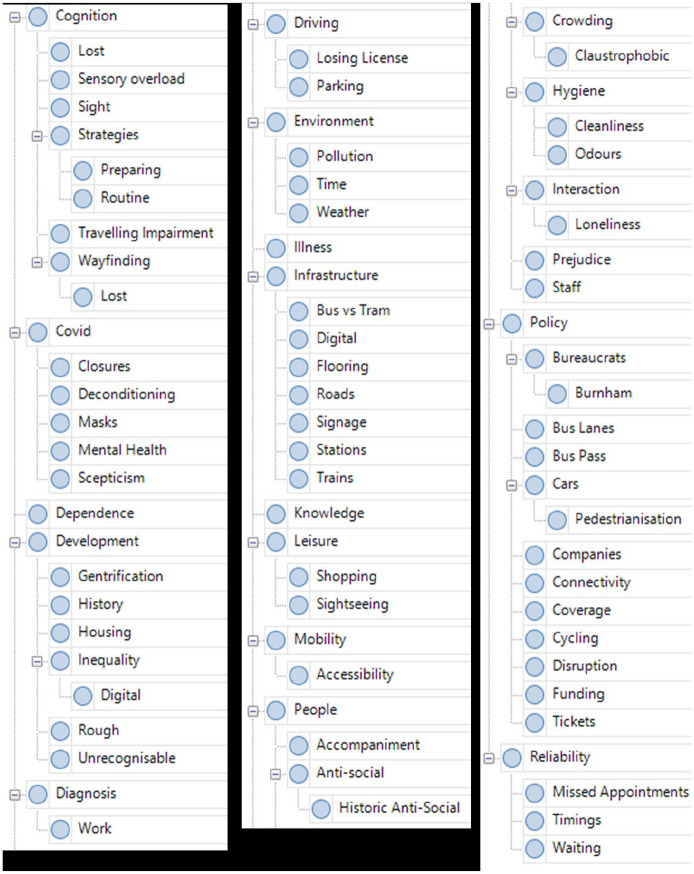
Coding scheme.

While transcribing the main audio file for a journey, I demarcated specific sections of audio that I subsequently deconstructed into smaller soundscape and quote clips using the Audacity programme. This meant that each journey had a corresponding collection of small audio files for use when mapping (returned to shortly), as well as the overarching audio file from the entire trip. Once I had the finished transcripts, I coded them using the NVivo software package using a crude and conventional thematic approach. By this, I mean that I read iteratively through the transcripts in NVivo, applying the codes and sub-codes that had seemed relevant to me during transcription (I noted all of these down) as well as those that occurred to me during this coding. These codes were all words or short phrases that conveyed something of the meaning of the section to which they were applied. I view this type of coding process more as a practice of data management than manifesting any great analytic value in its own right. However, as I will keep emphasising, the distinction between management and analysis of data seems somewhat illusory. In practice, the resulting NVivo coding scheme (Figure 12) provided a practicable means of retrieving specific portions of the dataset while creating project outputs – that is, if I want to see all instances where crowding is discussed, I select ‘crowding’ and have my participant’s insights to hand – but it would be misleading to claim that it has not been more implicitly embroiled in making-sense of the data to some extent, however minor.

**Figure 13.**
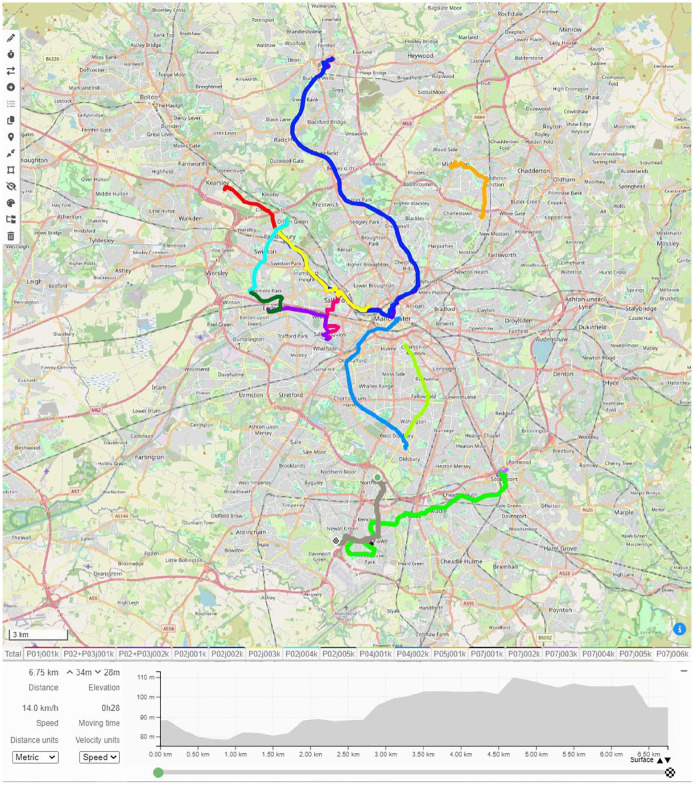
GPX visualisation.

Once downloaded, transcribed, coded, segmented, rendered, and filed, I began to map the journey data using the ATLAS.ti analysis software, which offers a broad suite of tools for working with maps and multimedia. The map-making process began with the creation of basic route maps based on the geolocation data. Unhelpfully, while ATLAS.ti does allow the analyst to work on accurate interactive maps in the form of GeoDocuments (these use the opensource OpenStreetMap platform), it does not facilitate the direct importation of GPX files, the most common format for geolocation recording.^1^ Therefore, I first had to create my maps by importing the Komoot-recorded GPX files into the GPX.studio platform, which generated a familiar visual route map (Figure 13). GPX.studio offers the same OpenStreetMap format that ATLAS.ti employs, aiding compatibility, and it allowed me to overlap journeys and scroll through them in real time. This interactive spacetime visualisation formed the blueprint for creating my more richly detailed ATLAS.ti map, because it enabled to me to cross-reference the time and location metadata attributed to the photograph and video files.

I decided to develop one GeoDocument for the entire project, rather than creating separate GeoDocuments for each journey or for each participant. This was an important decision that I agonised over for some time, and one that I only resolved following several journeys (which was regrettable given how much data had amassed by the time I made my mind up). My main rationale for pursuing a single all-encompassing map was that it became apparent during data generation that many seemingly poignant aspects of the participants’ experience were intimately bound up with Greater Manchester as a socio-material and political economic entity. Beyond the immediate minutiae of discrete infrastructures and happenings (e.g. a wet bus seat, a chatty bus driver), Greater Manchester in the singular loomed large in participants’ lives, and so I decided to have one overarching map that represented all participant journeys. Hence, the basic nature of the map came to represent one of the research findings, that is, the presence of the metropolis itself in conjuring more particular connective energies, and the layering of participant lives as functions of that connectropolis.

**Figure 14.**
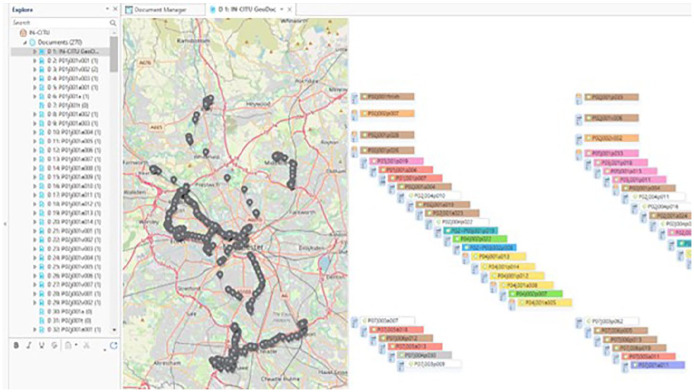
GeoDocument.

I primarily built up the maps through ATLAS.ti’s document, code, and quotation functions. I began by uploading all the documents relating to a given journey, namely the main audio file and transcript, the smaller audio clips of soundscapes and participant accounts with their respective transcripts, and the videos. I then coded the main project GeoDocument in reference to these multimedia documents. I first coded the start and finish points of journeys (coded as ‘P01j001start’ and ‘P01j001finish’), followed by codes for each audio clip, photograph, and video clip, corresponding with their unique identifier in the project key (Table 2). Each of these codes appears as a distinct pin on the GeoDocument in ATLAS.ti. This pinning of codes enabled me to visualise each journey in reference to its respective multimedia content, tracing out the GPX visualisation. Quotations can be added to codes, and besides text, these quotations can include image files. I used this function to attribute transcripts to audio codes and to upload the photographs directly to their respective codes on the map. Hence, if you click on a code pin, you can see the photograph captured at that point. Finally, I grouped the documents and codes for each journey and set a dedicated colour for each to distinguish individual journeys when viewing the entire GeoDocument. The result was an interactive journey map covering Greater Manchester, encompassing 270 documents, 684 codes, and 922 quotations (Figure 14). A word of warning – journeying analysis on this scale demands considerable storage and processing capacity, which can be an unfamiliar challenge for qualitative researchers. Your two-year-old university laptop (probably) will not suffice.

This map-making approach to the dataset, which I characterise as ‘journeying analysis’, was exploratory and therefore refined throughout the project. I began with ATLAS.ti’s basic capabilities and experimented, using them in different ways until I developed an approach that satisfied my purposes. Namely, I required an analysis approach that allowed me to synthesise all data forms, from geolocations to soundscapes, in a coherent and intuitive format that captured their place, motion, and connection. I would argue that this experimentation was a form of creative methodology, exemplifying the potentials for familiar software to furnish creative sociologies, particularly when conducting data analysis, by using them in ways perhaps not originally envisioned. For instance, my inability to import GPX data directly into ATLAS.ti led to my refashioning the other data forms to emulate geolocation data, inadvertently but aptly realising the multifaceted capacities for something like a collection of photographs to represent a journey photographically, as research data and in more surprising ways, hinting at the mobilities from whence they came.

The core praxis of journeying analysis is map-making, compiling a vast collection of written, verbal, soundscape, photographic, videographic, and geolocation data into something that has a meaningful journey-like quality. The map-making process is effectively a particular analogue of the general meaning-making process that typifies data analysis more broadly. Indeed, cartography has a rich history as a means of data analysis in social science and is especially suited to examinations of dynamic spaces as people navigate them. The act of map-making sensitises the analyst to the relations between and within the spaces mapped, and ultimately produces comparative visualisations of overlapping and intersecting, essentially *connecting*, data. Perhaps the most famous example of such a connective mapping approach in everyday life was John Snow’s cholera map of 1854, the map itself making palpable an otherwise intangible form of life (and death) (Snow, 1855). Beyond static visualisations of place, digital map-making based on geolocation data can also engage with and blend time (and by extension motion, i.e. place × time) into the analytic process by retracing an interactive version of the journey place by place, time by time. Hence, map-making can offer useful strategies for invigorating critical understandings of dynamic ecologies. As an example of this revelatory map-making, I quickly became aware of the spoke-hub distribution of much travel through Greater Manchester, with participants almost always travelling directly in to or out of city and town centres, even when travelling between two relatively proximate locations in the regional outskirts. This phenomenon (which is indicative of long-term privatisation having culled more circuitous routes, particularly those serving poorer communities) was rarely discussed explicitly, but, somewhat like cholera, the map-making process rendered it palpable.

It is worth noting that journeying analysis is rather heavy on data management. This is perhaps unsurprising given that I find great value (and, as mentioned, comfort) in blandly labour-intensive work with data, as opposed to approaches that celebrate a defter (and perhaps smarter) approach to extracting the meaning out of a dataset. I am unconvinced by traditional descriptions of coding, which perpetuate an idea of concentrating meaning down to some sort of explanatory core. Such meaning concentration seems at odds with the flux of connective energies and the holism that is integral to studying cognition ecologically. I treated coding, like transcription and the wider activities of map-making, as a dual process of management and analysis. First, that management – cataloguing, filing, depositing, compiling, and linking – was pragmatically essential to producing a dataset that it was possible for a mere mortal to engage with. Like any other person, I cannot hope to hold its unmanipulated entirety in my mind palace. Second, the doing of this management functioned methodologically as a sort of meditative praxis. I describe it as meditative because it is reminiscent of the peculiar interplay of mundanity, boredom, repetition, concentration, and effort that can typify meditation. It can force upon the analyst a peculiar affinity with the dataset, an affinity within which the interpretative art of sociological analysis happens. To this end, I documented my observations throughout the process to produce a single master text of bullet pointed hunches, linked to corresponding supporting data, that effectively told the story of the journeying analysis and the dataset upon which it was practised. This master document hence contained the story that I had to disseminate. That story (AKA ‘findings’) is well beyond the scope of this methodological article and is not told here, but it can be found in other articles (Fletcher, 2024, 2025).

## Possible destinations

An under-planned ethnography has an inherent vagueness to it. This generates important questions about the nature of data in such a project. Coming from a rather traditional interview-based methodological background, I was struck by the sheer scale of the data that I was collecting during each journey. This led me to reflect on the question of what I actually consider to be data, which itself morphed into what I thought data was doing in this research and other projects like it. When I use the word ‘doing’, I am keen to avoid indulging any notions of data as outrightly agentic, without recourse to a human investigator who orchestrates the research process. An excessive attribution of agency to data itself typifies appeals to new empiricism and post-qualitative research. Here, data are sometimes represented as having made its way to the researcher and then revealed the meanings within itself, with the researcher almost passively receiving the data’s wisdom (Aagaard, 2022). I am not convinced that this is a helpful approach for cognitive sociologies to take, despite the ontological turn resonances that I emphasise. From a practical perspective, it is improbable that data can be discretely agentic in social research, at least in a sense that can be articulated among humans by a human researcher. Moreover, a commitment to data as intrinsically agentic also fails conceptually, because leaning too hard into ‘things’ as doing their own things and letting them speak for themselves is at odds with monist ontologies that prioritise relationality. In every instance of research, I am a vital part of the ecology being researched, and my influence extends throughout it and the corresponding data. To suggest otherwise is suspect (Petersen, 2018).

One of the common post-qualitative data claims that I find more appealing is that the research is anti-representational (explicitly not ‘unrepresentative’). It flatly rejects the claim that sociological findings are somehow concretely extendable to the broader phenomena of which that empirical portion is contextualised as a portion of. Instead, data speak of the specific and the possible – a video of a bus journey is rather specific, yet it has some possibility of being recognised as a piece of ‘dementia research’ or ‘cognitive sociology’ or ‘social life’ or ‘art’ as it were, transcending its specificities (Petersen, 2018). Resonating with such post-qualitative sentiment, MacLure (2013) similarly dismisses representation in cognitive research, precisely because of its reliance on cognitivist notions of cognition, thought, and the mind that typify mainstream cognitivism, and which post-cognitivists therefore tend to refute. In a similar manner, Mason (2018) argues that connections are ineffable, that is, they exceed our abilities to articulate them, especially via linguistic communication. Importantly, ineffability is not the same as unknowability, so sociologists should not give up. Instead, their challenge is finding creative ways to bring audiences closer to an appreciation of the subject phenomena. The intersection of these post-qualitative, post-cognitivist, and connective dispositions is hence an anti-representational and pro-possibilities approach to telling research stories that evoke in audiences some sense of the connections at stake.

With this in mind, I would argue that an interactive multimedia map comprising various data forms generated with and by participants is a fine way of telling stories, as are smaller snippets of that multimedia. The combination of creative and digital methods opens up new forms of articulation – specifically via audio, photography, and videography – with which we can get around the constraints of language and writing, particularly when working with people for whom language might pose challenges. Any person familiar with any art form will likely recognise the potential for the more abstract non-linguistic communication of the ineffable. Images can affect us ineffably. Seemingly inconsequential images – for example, a photo of an ambiguously coloured dress posted to social media – can have a profound capacity for idiosyncratic reverberations, amplifying connections, and their repercussions. For this reason, an anti-representational approach can productively equivocate and leave possibilities open for the audience to navigate, solidify, and redefine as they engage with the work. Such wiggle room is necessary because the specificity of language (e.g. in findings papers) tends to concentrate meanings down, rather like a form of coding, in a manner that is alien to the study subject, particularly for those of us professionally trained to write in a pseudo-scientistic style centring on ideals of accuracy and precision.

Of course, it is easy to appreciate the power of creative and digital media to communicate the ineffable and evoke connection, but it is far more difficult to wield such power. I, like many social scientists, am no artist. I lack the skills to paint a picture, direct a film, or programme an app that will do justice to the phenomena that I research. Recognising this significant shortcoming, I again take inspiration from Mason’s assertion that ineffable subject matter should not preclude social scientists working to convey their research to audiences. The improbability of me emulating Van Gogh should not prevent me from attempting artful forms of articulation, just as the unlikelihood of emulating more talented scholars has not prevented me from publishing written work. Ultimately, what matters most is a commitment to continued effort, despite an awareness that one can never totally succeed, and an openness to diverse forms of articulation, accepting that others are more adept at those forms, and nobody is adept enough. For this project, I sought to implement a diverse pro-possibility approach to articulating the ineffable in several forms. Beyond writing, I am curating soundscape tracks, a photography exhibition, a film, and the map, all of which will be made publicly available. Together, these different forms of articulation offer a wide range of possibilities for audiences to engage with in their own ways as a means of developing their own appreciations of connective cognition. Ultimately, I intend this work to change the way people feel about cognition and place, and people with different cognitive experiences, and perhaps even to alter the ways in which they act in response. In doing so, my role as researcher has been to immerse myself in connective cognition and to translate that immersion into different resources for audiences to engage with.

Petersen (2018: 13) asks: ‘Wouldn’t a post-representational practice foreground the researcher’s ontologising activity rather than retreat from it, or even actively disaffirm it?’ It is a largely rhetorical question (Petersen’s answer is a firm ‘yes’) and one that reflects my experience. I am ever-present as the facilitator and curator at the heart of IN-CITU. The ontological turn, and a corresponding diversification of engagements with material worlds, does not neatly lead us into some radically new and improved epistemethodological space. Instead, it provides a means of reconceptualising our generation of and work with data. The question becomes – what can I do as the researcher, and hence one of, if not the key player in this situation, to make the data more conducive to possibility?

## Notes

1. I have spoken to the ATLAS.ti team about the GPX incompatibility and they do plan to address it.
